# Improving TB Case Detection Through Active Case-Finding: Results of Multiple Intervention Strategies in Hard-to-Reach Riverine Areas of Southern Nigeria

**DOI:** 10.9745/GHSP-D-23-00164

**Published:** 2024-02-28

**Authors:** Joseph N. Chukwu, Cosmas Kenan Onah, Edmund Ndudi Ossai, Charles C. Nwafor, Chukwuka Alphonsus, Okechukwu E. Ezeakile, Ngozi Murphy-Okpala, Chinwe C. Eze, Obioma Chijioke-Akaniro, Anthony Meka, Martin I. Njoku, Francis S. Iyama, Ngozi Ekeke

**Affiliations:** a German Leprosy and Tuberculosis Relief Association, Enugu, Nigeria.; b Department of Community Medicine, Alex Ekwueme Federal University Teaching Hospital Abakaliki, Abakaliki, Nigeria.; c Department of Community Medicine, Ebonyi State University, Abakaliki, Nigeria.; d National Tuberculosis, Leprosy and Buruli Ulcer Control Program, Abuja, Nigeria.

## Abstract

**Background:**

A major challenge to TB control globally is low case detection, largely due to routine health facility-based passive case-finding employed by national TB control programs. Active case-finding is a risk-population-based screening approach that has been established to be effective in TB control. This intervention aimed to increase TB case detection in hard-to-reach areas in southern Nigeria.

**Methods:**

Using a descriptive cross-sectional design, we conducted implementation research in 15 hard-to-reach riverine local government areas with historically recognized low TB case notification rates. Individuals with TB symptoms were screened using multiple strategies. Data were collected quarterly over a 4-year period using reporting tools and checklists. Descriptive analysis was done with Microsoft Excel spreadsheet 2019.

**Results:**

A total of 1,089,129 individuals were screened: 16,576 in 2017; 108,102 in 2018; 697,165 in 2019; and 267,286 in 2020. Of those screened, 24,802 (2.3%) were identified as presumptive TB, of which 88.8% were tested and 10% were diagnosed with TB (0.23% of those screened). TB notifications more than doubled, increasing by 183.3% and 137.5% in the initial implementation and scale-up, respectively. On average, 441 individuals needed to be screened to diagnose 1 TB case. The cases, predominantly males (56.1%) and aged 15 years and older (77.4%), comprised 71.9% bacteriologically confirmed drug-sensitive TB, 25.8% clinically diagnosed drug-sensitive TB, and 2.3% drug-resistant cases. Detection sources included community outreach (1,786), health facilities (505), people living with HIV (57), and household contacts of bacteriologically confirmed TB cases (123). Remarkably, 98.1% of diagnosed TB cases commenced treatment.

**Conclusions:**

We found a significant yield in TB case notifications, more than doubling the baseline figures. Given these successful results, we recommend prioritizing resources to support active case-finding strategies in national programs, especially in hard-to-reach areas with high-risk populations, to address TB more comprehensively.

## Abstract (precis)

To address TB control efforts more comprehensively, active case-finding is another important approach to increasing TB case detection rates, especially in hard-to-reach areas with high-risk populations.

## Teaser

**Key Findings:**

- Compared to passive case-finding, active case-finding (ACF) is an important approach to increasing case detection rates by identifying individuals with TB who have no symptoms and improving access to diagnosis in hard-to-reach areas.
- Implementing this ACF intervention in these hard-to-reach areas led to a substantial increase in TB case notifications by 183.3% and 137.5% during initial implementation and scale-up, respectively, suggesting the effectiveness of the ACF intervention in identifying previously undiagnosed cases.
- This significant increase in case detection emphasizes the urgency for heightened attention, more efficient resource allocation, and use of intervention strategies to address the low TB case notifications in Nigeria, especially in hard-to-reach riverine areas with high-risk populations.

**Key Implications:**

- Health policymakers and TB control program managers could prioritize resources toward ACF for TB if, compared to other potential uses of resources, ACF is more cost effective.
- TB control program managers could implement more and regular ACF interventions for TB in hard-to-reach areas, especially in high TB-burden countries, to boost TB case detection and facilitate the efforts toward ending TB.

## INTRODUCTION

Worldwide, TB remains a major global health problem and a leading cause of death.^1^ Every day, close to 4,000 people die from the disease, and about 30,000 develop active disease.^2^ In 2021, the World Health Organization estimated that of the 10.6 million who fell ill with TB globally, 1.4 million people died of TB.^3^ Although TB is globally declining at an annual rate of 1.5%–2%, the rate of decline is unlikely to align with the End TB Strategy, which targets a 90% reduction in TB deaths and an 80% reduction in TB incidence by 2030 compared to 2015 levels.^4^ Despite the existence of effective diagnostic tools, a major challenge to TB control is low case detection.^5^ From 2018 to 2021, only 26.3 million estimated TB cases were treated, amounting to 66% of the target of 40 million for 2018 to 2022.^3^

Nigeria is a high TB burden country that accounted for 4.6% of the global disease burden in 2020 and 4.4% of the incident TB cases globally in 2021.^3^^,^^6^ Nigeria is ranked seventh among the 30 high-burden countries for TB, HIV-associated TB, and multidrug-resistant TB in the World Health Organization list for the period from 2021 to 2025.^6^^,^^7^ TB accounts for more than 10% of all deaths, and every hour, nearly 30 people die from the disease in Nigeria.^7^ Despite the huge TB burden in Nigeria, the country has one of the lowest TB case detections globally.^5^^,^^8^^–^^13^ With an estimated population of about 226.2 million,^14^ Nigeria is estimated to have more than 440,000 incident TB cases, and over 300,000 are estimated to be missed annually.^15^ As of the fourth quarter of 2022, the notification rate in Nigeria stood at 79,048 cases.^16^ In 2021, the country contributed 6.3% to the global gap between estimated TB incidence and the reported number of people newly diagnosed with TB and was among the 10 countries that accounted for about 70% of the global gap between the estimated global incidence of multidrug-resistant/rifampicin-resistant TB and the number of people enrolled in treatment.^3^ Nigeria accounts for 4% of the world’s burden of drug-resistant TB (DR-TB) and the highest burden (27% of the incidence) in Africa.^12^^,^^17^^,^^18^

Despite the huge TB burden in Nigeria, the country has one of the lowest TB case detections globally.

Nigeria’s National Tuberculosis, Leprosy, and Buruli Ulcer Control Program (NTBLCP) relies largely on passive case-finding (PCF) to identify people with TB, a measure that only identifies individuals with TB who present with symptoms at health facilities.^19^ A TB prevalence survey showed that more than 70% of TB cases had no reported symptoms but had an abnormal chest X-ray, showing that passive, health facility-based case-finding alone is inadequate for assisting national programs to reach the End TB Strategy targets.^4^ A unique challenge to PCF in hard-to-reach riverine areas of Nigeria is poor accessibility to health facilities. The challenging riverine areas of Nigeria’s Niger Delta are characterized by intricate water networks and dense vegetation and pose unique obstacles to conventional transportation and development. Navigating through the Niger Delta areas demands specialized means such as boats, with lush riverbank vegetation further complicating access. Despite the challenges, these remote regions are home to diverse communities with distinct cultural identities and significant ecological value. The poor access of the communities in these areas often results in the underdiagnosis of TB.

Active case-finding (ACF) is defined by the World Health Organization as systematic screening for active TB, normally outside of health facilities, but could also be undertaken at health facilities in a targeted population considered at higher risk of developing TB,^19^ employing approaches, such as community mobilization, contact tracing, and house-to-house screening, to reach undiagnosed individuals not presenting to health facilities. ACF is an important approach in hard-to-reach areas where access to health facilities for PCF is limited and is an indispensable approach to attaining the 2035 End TB Strategy targets.^4^

To increase TB case detection, the German Leprosy and TB Relief Association (GLRA) implemented ACF interventions in 15 selected hard-to-reach riverine local government areas (LGAs) with historically recognized low TB case notifications in 6 states of southern Nigeria. This ACF intervention aimed to increase case detection, and the target was to double TB case notifications in the affected areas.

## METHODS

The intervention was implemented by GLRA, a nonprofit, nongovernmental organization with the core objectives of providing care and support to people affected by leprosy, TB, and disabilities through medical treatment and social rehabilitation. Funded with a grant awarded by the Stop TB Partnership’s TB REACH initiative, the project, TB REACH Wave 5, and its scale-up, “Community-Driven Output-Based-Approach to TB Service Delivery in Hard-to-Reach Riverine Areas of Southern Nigeria,” were implemented between January 2017 and August 2020. TB REACH is an annual Stop TB Partnership initiative in which relatively small grants are given to execute innovative, scalable TB control projects for a period lasting between 12 and 18 months. The idea is to provide a template that can be scaled up through better-resourced funding mechanisms or organizations like the Global Fund. This TB REACH (Wave 5) was funded by the Bill & Melinda Gates Foundation and the Canadian Government.

### Study Areas

**FIGURE 1:**
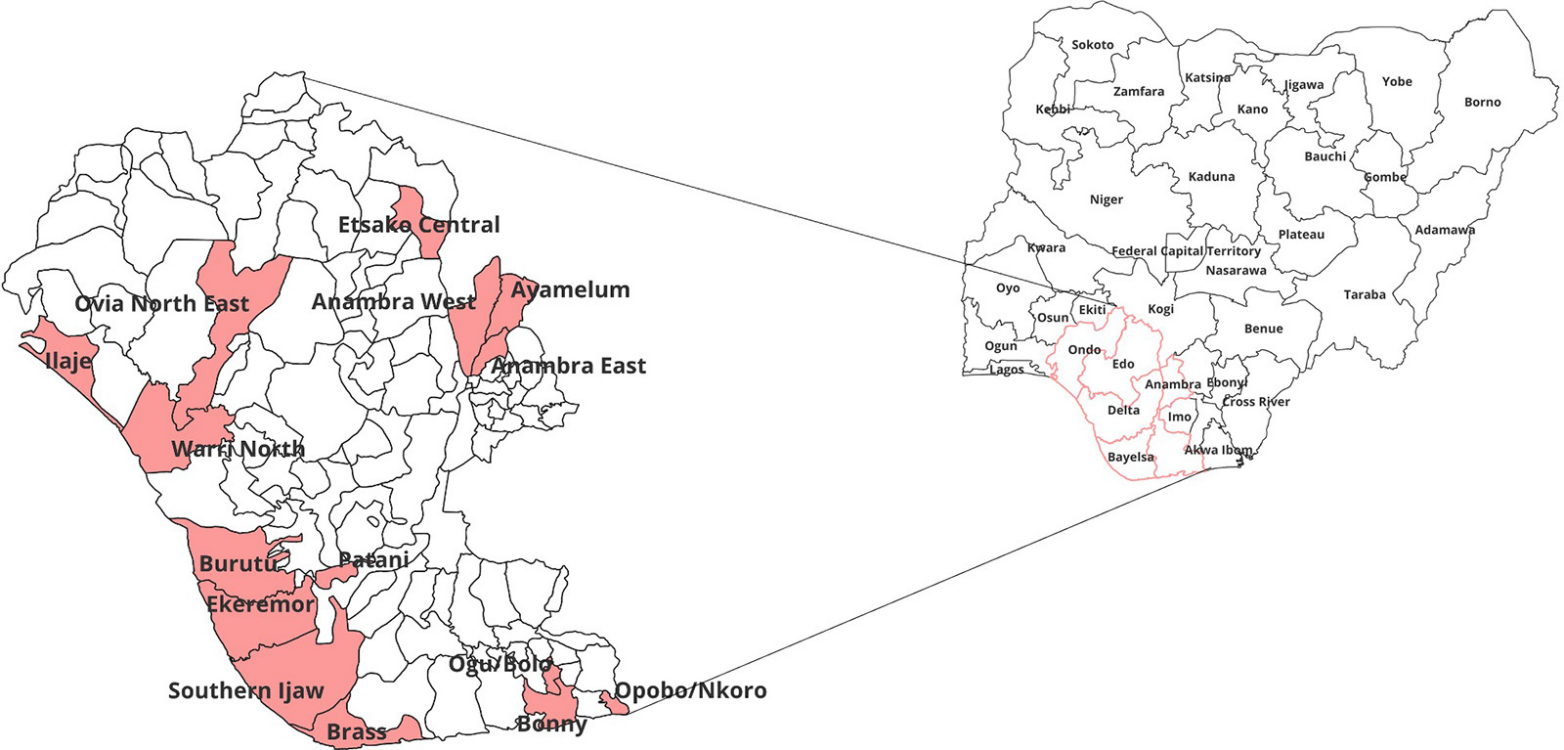
Map of Nigeria Showing Intervention Areas for Active Case-Finding for TB Implemented in 15 Hard-to-Reach Riverine Local Government Areas Across 6 States of Southern Nigeria

The intervention was implemented in 15 hard-to-reach riverine LGAs in 6 states in southern Nigeria: Anambra East, Anambra West, and Ayamelum LGAs in Anambra State; Brass, Ekeremor, and Southern Ijaw LGAs in Bayelsa State; Warri North, Burutu, and Patani LGAs in Delta State; Ovia North East and Etsako Central LGAs in Edo State; Ilaje LGA in Ondo State; and Bonny, Ogu-Bolo, and Opobo Nkoro LGAs in Rivers State (Figure 1). These LGAs were purposively selected because they are hard-to-reach riverine areas and are historically recognized for low TB case notifications. The notification rate for TB in these LGAs ranged between 2 and 21, with an average of 7 per quarter of reporting per LGA. During the mapping of each LGA, a comprehensive listing of all the communities within them was also done.

This intervention was implemented as the initial phase and the scale-up. The key difference between the initial TB REACH Wave 5 and the scale-up lies essentially in scope and location. The initial project spanned the 6 LGAs in Bayelsa and Delta States with a cumulative population of 1,599,140. The scale-up extended the project to an additional 9 LGAs in Anambra, Edo, Ondo, and Rivers States with a cumulative population of 2,027,290, making a total of 15 LGAs in 6 states with an aggregate population of 3,626,430. This intervention aimed to double the TB case notifications in these LGAs by the end of the project.

### Study Design and Population

This implementation research used a descriptive cross-sectional design. The population studied are individuals who exhibited signs and symptoms of TB. This included individuals with a cough lasting for several weeks, chest pain, weight loss, fever, and night sweats.

### Intervention Strategies

The intervention was conducted under routine program implementation by integrating the intervention into the existing national TB program, using sample sputum cups and laboratories, and the reporting structure, in line with the established protocols. To achieve the desired outcome, 4 intervention strategies were applied: targeted community outreach, health facility-based screening, screening among people living with HIV (PLWH), and screening among household contacts of bacteriologically confirmed (Bac+) TB cases. Operationally, eligibility for screening in all the intervention strategies was defined as any individual with a cough lasting up to 2 weeks, unexplained weight loss, fatigue, contact with a TB patient, and/or fever. Additionally, PLWH on treatment with a cough of 1 week, in addition to weight loss and/or fever, were considered due to their higher risk status. Any children who were regular close contacts of confirmed TB cases, had persistent rapid breathing, and/or poor weight gain or failure to thrive were considered, independently of the other stated qualifying criteria. In the community outreach approach, chest camps, schools, and religious settings were included. The facility screening was done in private hospitals and public primary and secondary health facilities in the LGAs that hosted antenatal care, child health, and HIV clinics during every hospital visit. Contacts of Bac+ TB were traced to their homes for screening.

#### Human Resources and Capacity-Building

A key innovation in this project was the engagement of volunteer community members as designated volunteer liaison officers (VLOs), who were unemployed male and female graduate youths residing in and familiar with the intervention areas and owned a smartphone. Community gatekeepers and leaders played key roles in identifying those who were selected, interviewed, and engaged as VLOs to foster ownership and sustainability in the communities.

Under the supervision and guidance of GLRA and the TB and leprosy supervisors, the VLOs were trained to conduct TB screening, sample collection, sample shipment, completion of necessary registers and forms, contact tracing and investigation, and linkage of positive TB cases to local directly observed treatment short-course (DOTS) providers for initiation of TB treatment. VLOs in all the LGAs received a monthly stipend to maintain linkages with various communities and ensure that all patients had access to TB services and were paid an allowance based on the number of TB cases diagnosed to boost their productivity.

Community and health facility TB workers were engaged and local clinicians and medical officers were trained and retrained on the diagnosis of TB in children, as well as in adults, with negative GeneXpert MTB/RIF (*Mycobacterium tuberculosis* and rifampicin-resistant TB strains) assay results using chest X-rays (CXRs) and clinical examinations. To ensure quality assurance in childhood TB diagnosis, a community of practice comprising program managers, childhood TB focal persons, pediatricians, local clinicians, and GLRA technical staff was established using a dedicated WhatsApp platform.

#### Screening, Testing, Diagnosis, and Treatment

In all 4 intervention strategies, individuals were screened to identify presumptive TB cases defined as having the following TB-like symptoms: cough of up to 2 weeks duration, hemoptysis, night sweats, weight loss, chest pain, fatigue, contact with a TB patient, and/or fever. Sputum samples were collected from those who qualified as a presumptive TB case. During the community outreach approach, samples collected from presumptive TB cases were often sent to either TB microscopy or GeneXpert MTB/RIF sites through water transport. Most of the GeneXpert sites were already established public laboratories. However, the project also procured and installed 2 GeneXpert machines within existing public facilities that had no such machines before. Where GeneXpert MTB/RIF test was unavailable, samples were redirected to TB microscopy sites for testing, which is consistent with national guidelines.

Clinical diagnosis of cases was also carried out in individuals with an unproductive cough who could not produce sputum specimens for laboratory diagnosis but had sufficient clinical evidence of TB. Most of the clinically diagnosed childhood TB was done using CXR. Free CXR services were available in a few laboratories funded by the Global Fund within the TB control program. However, they were underutilized due to the travel cost associated with accessing the distant services. The project trained clinicians to aid in interpreting the CXR and facilitated the testing by covering the cost of transporting the children to the CXR facilities.

The VLOs and community and health facility TB workers were regularly supervised by GLRA technical staff and state program staff, as well as LGA TB and leprosy supervisors, who regularly visited predetermined diagnostic health facilities, which serve as DOTS sites. During each visit, the TB and leprosy supervisors checked all reported TB cases and compiled the data for the quarterly surveillance report, which was sent to the state and national programs. Furthermore, additional supervisory visits were conducted regularly by the state program team in conjunction with the GLRA technical team to perform random checks on reported activities and verify compliance with the project’s operational procedures. Due to the riverine nature of the intervention areas and the high cost of water transport, remote monitoring and supervision were also undertaken by the GLRA technical team using a mobile phone.

Upon diagnosis, TB cases were reported to the appropriate TB and leprosy supervisors, who facilitated discussions between the patients, health care providers in DOTS facilities, and the VLO responsible for identifying the TB case. These discussions aimed to determine the most suitable and closest DOTS facility for monitoring the patient’s TB treatment, particularly during the second, fifth, and sixth months of treatment. The majority of the TB cases diagnosed were started on treatment at DOTS facilities. From this point, TB cases were managed through DOTS services by regular government health care workers trained in TB treatment according to the national treatment guidelines.

Under the directives of the TB and leprosy supervisors, the VLOs played vital roles in ensuring treatment completion. Their responsibilities included notifying about drug stock-outs and providing support for transportation and logistics involved in medicine delivery, especially in critical, unsafe, or challenging situations. Additionally, the VLOs maintained regular communication with patients and addressed their treatment welfare concerns, particularly in remote areas, as part of their duties.

Over 1 year, the VLOs conducted ACF activities twice a week in the communities until complete coverage of the LGA was achieved. Areas deemed as hotspots or high-risk communities were revisited multiple times for intensified ACF efforts. In addition, the VLOs collaborated with community gatekeepers to identify and engage other community volunteers who played crucial roles in ongoing community sensitization and in collecting and transporting samples, especially after the scheduled VLO visits.

The project adopted an output-based approach for incentives to the relevant stakeholders within the communities, allocating a total of 22,000 Nigerian naira (N) (US$28.50) per TB case diagnosed. This conditional cash payment was disbursed upon the successful accomplishment of specific milestones. The distribution of this incentive is as detailed: N5,000 (US$6.47) to the referring community member; N5,000 (US$6.47) to the ward development committee for the purchase of hospital or care-related items within the community; N4,000 (US$5.17) to the health facility responsible for managing the diagnosed case; N4,000 (US$5.17) to the health care worker responsible for overseeing the completion of the treatment; N2,000 (US$2.59) to the laboratory facility that conducted the diagnosis; and N2,000 (US$2.59) to the ad-hoc laboratory focal person involved in the diagnostic process. To ensure quality control, payments were contingent upon the achievement of the desired outcomes. Notably, no payment was issued in the absence of the expected results. This strategy aimed to maintain the integrity of the entire care cascade, recognizing the interconnectedness of various components in the service delivery process.

A signed informed consent form was provided by all the study participants after they were adequately informed of the project’s purpose. Before signing the informed consent form, all the participants were assured that the confidentiality of their personal information would be maintained. All data were collected without any participant identifying information.

### Variables and Sources of Data

Several key variables were considered to assess the reach of our intervention (Box). The data for these variables were obtained from a variety of sources, including ACF screening forms, presumptive TB registers, laboratory registers, and TB treatment registers.

BOX Variables Assessed in an Active Case-Finding Intervention to Improve TB Case Detection in Hard-to-Reach Riverine Areas of Southern Nigeria

- **Number of individuals screened for TB**: The total count of individuals who underwent TB screening within a defined period.
- **Number of presumptive TB identified (% of those screened)**: The number of individuals screened for TB who were flagged as presumptive TB cases and the proportion of all those screened based on initial assessments and symptoms.
- **Number of presumptive TB tested (% of presumptive TB identified)**: The number of presumptive TB cases that underwent further diagnostic testing, expressed as a percentage of the presumptive cases identified.
- **Number of people with all forms of TB diagnosed (% of presumptive TB identified)**: The number of individuals among the presumptive TB cases who received a confirmed diagnosis of TB, encompassing all its forms, and the percentage of the presumptive cases identified.
- **Number of bacteriologically confirmed drug-sensitive TB (Bac+ DS-TB) diagnosed (% of all forms of TB)**: The number of individuals diagnosed with Bac+ DS-TB and percentage of the total number of TB cases, irrespective of the TB form.
- **Number of clinically diagnosed DS-TB (% of all forms of TB)**: The number of DS-TB cases diagnosed through clinical assessments and evaluations, relative to all forms of TB diagnosed.
- **Number of DR-TB cases diagnosed (% of all forms of TB)**: The number of drug-resistant TB cases identified and percentage of the total TB cases diagnosed, regardless of the TB form.
- **Number of all forms of TB started on treatment (% of all forms of TB identified)**: The number of individuals diagnosed with any form of TB who initiated treatment, ensuring timely intervention, and percentage of the total TB cases identified, regardless of the TB form.

### Data Collection and Analysis

Individual-level data were collected for all those screened using the ACF screening form and collated via ODK every quarter from the third quarter of 2017 through the third quarter of 2020. The form was adapted from the national TB screening protocol and reviewed by experts in TB, including TB physicians and technical team members from the NTBLCP. Information captured in the form included signs and symptoms of TB, intervention strategy, age, sex, address, contact number, and an individual-specific code. All presumptive TB cases identified were additionally captured in a paper-based specimen/clinical examination request form that had the individual-specific code. A screenshot was taken of the specimen/clinical examination request form and shared on a designated WhatsApp platform as backup data. Sputum samples collected for testing were accompanied by the appropriately filled specimen/clinical examination request form to the DOTS facility, where the data were entered into the presumptive register and the laboratory register. Individuals who could not produce sputum were sent with their specimen/clinical examination request form to the clinicians for clinical and radiological examination. All confirmed TB cases were placed on treatment and documented in the TB treatment registers in DOTS centers and notified to the corresponding TB and leprosy supervisors.

Aggregate data were collated and tabulated in Microsoft Excel, disaggregated by quarter, sex, and age. The individual-specific number codes were used to match the test and treatment data collected from the TB registers with the individual-level data collated with ODK. After cleaning and organization, the data were analyzed with Microsoft Excel 2019 using the Excel AutoSum computation commands. Summary statistics were computed by intervention strategy, sex, quarter, and year of reporting.

### Ethical Approval

This project was reviewed and approved by the Health Research Ethics Committee of Delta State Ministry of Health, Asaba, Nigeria (reference number HC/218/VOL IV/134). Further, approvals were obtained from the NTBLCP and the State TB, Leprosy, and Buruli Ulcer Control Program of all the participating states.

## RESULTS

Table 1 shows the distribution of the participants by age, sex, and intervention strategy. A total of 1,089,129 individuals were screened for TB, of whom a majority were females (54.7%) and aged 15 years and older (53.0%). In terms of the intervention strategy, individuals screened at the community outreach constituted 81.8%, followed by those screened at the health facilities (15.6%).

**TABLE 1.** Distribution of Participants Screened for TB During Active Case-Finding Implemented in 15 Hard-to-Reach Riverine Local Government Areas in Southern Nigeria

**Variable**	**Frequency, No. (%)(N=1,089,129)**
Age, years	
0–4	164,583 (15.1)
5–14	346,980 (31.9)
≥15	577,566 (53.0)
Sex	
Female	595,391 (54.7)
Male	493,738 (45.3)
Intervention strategy	
Community outreach	890,475 (81.8)
Health facility	170,002 (15.6)
HIV-positive people	24,030 (2.2)
Household contacts of bacteriologically confirmed TB	4,622 (0.4)

Table 2 shows the change in the number of TB cases notified following ACF in the 15 LGAs where the intervention was implemented. Before the intervention, an average of 6 cases of TB were notified per quarter among 6 LGAs that participated in the initial TB REACH Wave 5. During the intervention, this number increased to 17, giving a percentage increase of 183.3%. Similarly, the average number of TB cases notified across the 9 LGAs that were included during the scale-up increased from 8 cases at baseline to 19 cases during the intervention, amounting to a percentage increase of 137.5%.

**TABLE 2.** Change in TB Notifications Following an Active Case-Finding Intervention in 15 LGAs Across 6 States in Southern Nigeria

**LGA**	**Population**	**Average No. of TB Notified per Quarter per LGA Before Intervention**	**Average No. of TB Notified per Quarter per LGA During Intervention**	**% Increase in No. of TB Notified**
		(Quarter 1 2014–Quarter 2 2017)	(Quarter 3 2017– Quarter 3 2020)	
Warri North	186,918	4	13	225.5
Burutu	285,531	6	12	100.0
Ekeremor	359,499	11	21	90.9
Southern Ijaw	429,135	5	23	360.0
Patani	92,521	2	10	400.0
Brass	245,536	8	24	200.0
**Total**	1,599,140	6	17	183.3
		(Quarter 1 2015– Quarter 4 2018)	(Quarter 1 2019– Quarter 3 2020)	
Anambra East	206,155	7	20	66.7
Anambra West	226,688	2	22	100.0
Ayamelum	214,289	11	26	62.5
Ovia North East	210,034	6	13	62.5
Etsako Central	127,402	5	15	400.0
Bonny	311,045	9	12	100.0
Ogu-Bolu	108,921	2	6	500.0
Opobo Nkolo	221,124	9	33	266.7
Ilaje	401,632	21	24	4.3
**Total**	2,027,290	8	19	137.5

Abbreviations: LGA, local government area.

The average number of TB cases notified increased during the initial implementation and scale-up.

Table 3 shows the number of individuals needed to screen to diagnose 1 case of TB in each of the various intervention strategies, age groups, and sex. On average, the number needed to screen to diagnose 1 case of TB was 441.

**TABLE 3.** Number Needed to Screen to Diagnose 1 Case of TB During an Active Case-Finding Intervention

**Variable**	**Number Needed to Screen**	
	**Per Group**	**Aggregate Index**
Intervention strategy		
Community outreach screening	499	441
Health facility screening	337	
Screening among HIV-positive people	422	
Household contacts of bacteriologically confirmed TB cases	38	
Age group, years		
0–4	596	441
5–14	1,230	
≥15	302	
Sex		
Female	548	441
Male	356	

**FIGURE 2:**
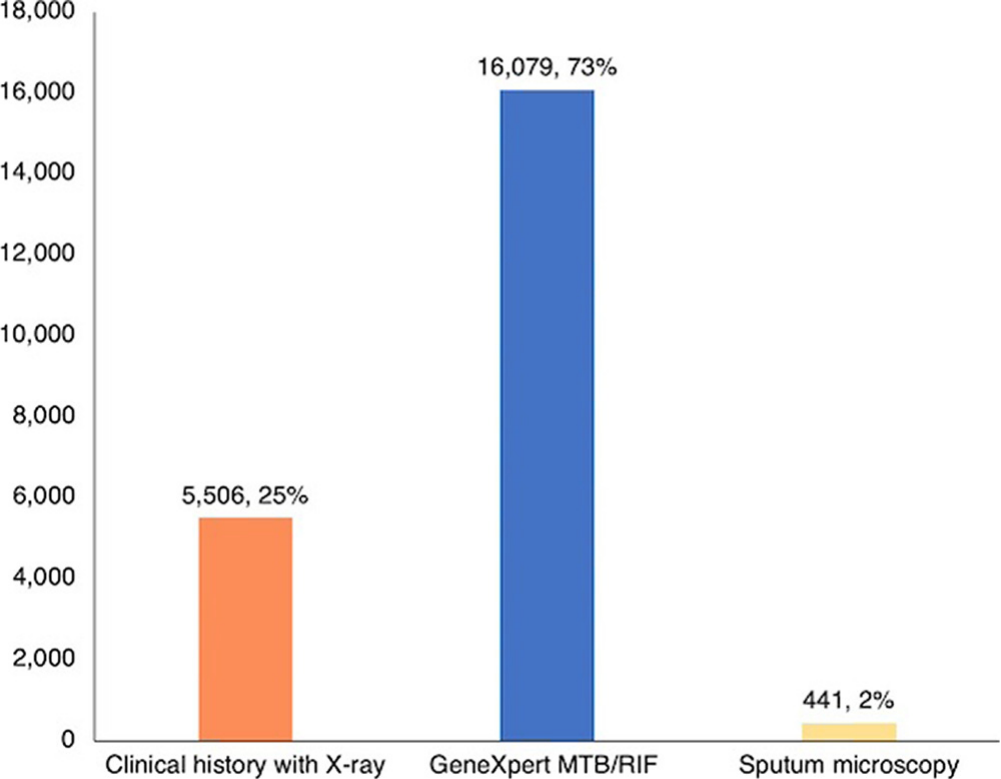
Method of Diagnosis During an Active Case-Finding for TB Intervention Implemented in Hard-to-Reach Riverine Local Government Areas in Southern Nigeria Abbreviation: MTB/RIF, *Mycobacterium tuberculosis* and rifampicin-resistant TB.

Table 4 shows the result of the interventions disaggregated according to age and sex distributions. Of those screened, 24,802 (2.3%) were identified as presumptive TB cases. Among the presumptive TB cases, 22,026 (88.8%) were tested for TB, and 2,471 (10%) were diagnosed with the disease. The TB cases diagnosed represent 0.23% of the individuals who were screened. Case notification increased by 183.3% and 137.5% in the initial implementation and the scale-up, respectively. Of the proportion of presumptive cases with diagnoses confirmed, there were more males (11.9%) than females (8.3%), and more of those aged 15 years and older (11.5%) compared to those aged 0–4 years (9.6%) or 5–14 years (5.4%) (Table 4). Of the cases detected, 1,776 (71.9%) were Bac+ DS-TB, 637 (25.8%) were clinically diagnosed with DS-TB, and 58 (2.3%) were DR-TB. Of all forms of TB diagnosed within each age group, Bac+ cases constituted a significant majority (86.3%) among those aged 15 years and older, and clinically diagnosed cases were the majority in those aged 0–4 years (84.1%) and 5–14 years (68.8%). Of the TB cases detected, 2,471 (98.1%) were started on treatment. A significant majority of the diagnoses were made using GeneXpert MTB/RIF (73%), and a quarter (25%) of the cases were diagnosed with clinical history supported with X-ray (Figure 2).

**TABLE 4.** Result of Active Case-Finding for TB Intervention Implemented in 15 Hard-to-Reach Riverine Local Government Areas in Southern Nigeria, Disaggregated by Age and Sex

**Performance Indicator**	**Total Output**	**Age Distribution, Years**			**Sex Distribution**	
		**0–4**	**5–14**	**≥15**	**Female**	**Male**
No. of individuals screened for TB	1,089,129	164,583	346,980	577,566	595,391	493,738
No. of presumptive TB identified (% of individuals screened)	24,802 (2.3)	2,883 (1.8)	5,213 (1.5)	16,706 (2.9)	13,126 (2.2)	11,676 (2.4)
No. of presumptive TB tested for TB (% of presumptive TB identified)	22,026 (88.8)	2,118 (73.5)	4,353 (83.5)	15,555 (93.1)	11,724 (89.3)	10,302 (88.2)
No. of all forms of TB diagnosed (% of presumptive TB identified)	2,471 (10.0)	276 (9.6)	282 (5.4)	1,913 (11.5)	1,086 (8.3)	1,385 (11.9)
No. of Bac+ DS-TB diagnosed (% of all forms of TB diagnosed within group)	1,776 (71.9)	42 (15.2)	84 (29.8)	1,650 (86.3)	765 (70.4)	1,011 (73.0)
No. of clinically diagnosed DS-TB (% of all forms of TB diagnosed within group)	637 (25.8)	232 (84.1)	194 (68.8)	211 (11.0)	294 (27.1)	343 (24.8)
No. of DR-TB diagnosed (% of all forms of TB diagnosed within group)	58 (2.3)	2 (0.7)	4 (1.4)	52 (2.7)	27 (2.5)	31 (2.2)
No. of all forms of TB started on treatment (% of all forms of TB diagnosed)	2,425 (98.1)	273 (98.9)	276 (97.9)	1,876 (98.1)	1,065 (98.1)	1,360 (98.2)

Abbreviations: Bac+, bacteriologically confirmed; DS-TB, drug-sensitive TB; DR-TB, drug-resistant TB.

Table 5 shows the result of the intervention disaggregated according to the strategy applied. Altogether, 890,475 individuals were screened in the communities, 170,002 in the health facilities, 24,030 among PLWH, and 4,622 among household contacts of Bac+ TB. A total of 1,786 TB cases (9.4%) were diagnosed in the communities, 505 (11.1%) in the health facilities, 57 (14.4%) among PLWH, and 123 (15.3%) among household contacts of Bac+ TB. Bac+ DS-TB dominated the TB cases across nearly all intervention strategies (community outreach: 72.6%, health facilities testing: 77.8%, and testing among PLWH: 73.7%) except household contacts of Bac+ TB, where a clinical diagnosis was highest (61.8%). The prevalence of DR-TB was highest (3.2%) among individuals whose samples were collected in health facilities and least (1.6%) among household contacts of Bac+ TB. The Supplement Table shows the result of the intervention per year from 2017 to 2020, disaggregated according to the intervention strategy. Across the 4 intervention strategies, the highest screening for TB and highest tests occurred in 2019. The highest case detection happened in 2020 among household contacts of Bac+ confirmed TB, but in 2019, it happened in all other intervention strategies.

**TABLE 5.** Result of Active Case-Finding for TB Intervention Implemented in 15 Hard-to-Reach Riverine Local Government Areas in Southern Nigeria, Disaggregated by Intervention Strategy

**Performance Indicator**	**Total Output**	**Output per Intervention**			
		**Community Outreach**	**Health Facility**	**People Living With HIV**	**Household Contacts of Bac+ TB**
No. of individuals screened for TB	1,089,129	890,475	170,002	24,030	4,622
No. of presumptive TB identified (% of those screened)	24,802 (2.3)	19,044 (2.1)	4,560 (2.7)	396 (1.6)	802 (17.4)
No. of presumptive TB tested (% of presumptive TB identified)	22,026 (88.8)	16,698 (87.7)	4,258 (93.4)	369 (93.2)	701 (87.4)
No. of people with all forms of TB (% of presumptive TB identified)	2,471 (10.0)	1,786 (9.4)	505 (11.1)	57 (14.4)	123 (15.3)
No. of Bac+ DS-TB diagnosed (% of all forms of TB)	1,776 (71.9)	1,296 (72.6)	393 (77.8)	42 (73.7)	45 (36.6)
No. of clinically diagnosed DS-TB (% of all forms of TB)	637 (25.8)	450 (25.2)	96 (19.0)	15 (26.3)	76 (61.8)
No. of DR-TB cases diagnosed (% of all forms of TB)	58 (2.3)	40 (2.2)	16 (3.2)	0 (0)	2 (1.6)
No. of all forms of TB started on treatment (% of all forms of TB diagnosed)	2,425 (98.1)	1,748 (97.9)	498 (98.6)	56 (98.2)	123 (100)

Abbreviations: Bac+, bacteriologically confirmed; DS-TB, drug-sensitive TB; DR-TB, drug-resistant TB.

## DISCUSSION

This ACF intervention more than doubled TB case notifications, with substantial percentage increases in quarterly reporting rate by 183.3% and 137.5%, respectively, in the initial TB-REACH Wave 5 and the scale-up LGAs. With the project’s estimated cost of N197,104,278 (US$254,776) and 2,471 cases of TB diagnosed, the cost per case identified is approximately US$103. With an average increase in case notification per quarter per LGA of 11 during the intervention, there were 1,551 more cases than otherwise identified through PCF. This significant increase in case detection suggests the effectiveness of the ACF intervention in identifying previously undiagnosed cases, emphasizing the urgency for heightened attention, more efficient resource allocation, and employment of workable intervention strategies to address and manage the protracted low TB case notifications in Nigeria, especially in hard-to-reach riverine areas with high-risk populations.

The period prevalence of TB in our study is 0.23%, which is approximately 4 times the national average for 2017–2020 (0.06%), estimated using the total TB cases notified^16^ and the projected populations of Nigeria over the same period.^14^ Although not unexpected, bearing in mind that our screening targeted high-risk populations in hard-to-reach areas, the higher prevalence in this study highlights that prioritizing and intensifying ACF efforts, specifically within these high-risk populations, is important for addressing TB more comprehensively and achieving effective control.

The higher period prevalence of 0.23% in this study highlights that prioritizing and intensifying ACF efforts, specifically within these high-risk populations, is important for addressing TB more comprehensively and achieving effective control.

The need for ACF among high-risk populations in hard-to-reach riverine areas of Nigeria is particularly critical, given the limited access to health facilities for PCF. This urgency is underscored by the fact that only 31% of the country’s health facilities provide TB treatment services.^20^ The limited coverage of TB treatment services contributes significantly to the high proportion of undetected cases. The effectiveness of ACF in TB case detection has been well-documented.^21^ Modeling studies indicate that ACF interventions tend to be cost effective.^19^^,^^22^ The cost per case detected may seem high, but this assessment should consider variables such as the prevalence of the condition, specific strategies employed, and contextual factors.

This is because the population-level benefits of ACF accumulate with time as additional cases are prevented through a halt of the transmission process. By reducing transmission and successfully treating a large proportion of TB cases, the incidence, prevalence, and mortality due to the disease will decrease.^22^ Prioritizing ACF in hard-to-reach areas may help improve the detection, treatment, and control of TB in these areas, thereby reducing the burden of the disease and its impact on the affected communities.

Over half of the identified cases were males (56.1%) and aged 15 years and older (77.4%). This observation aligns with findings from previous studies by Nwene and Jumbo et al., indicating a higher incidence of TB in males compared to females.^23^^,^^24^ The 2019 annual report of the NTBLCP also reported that 61% of cases identified were male,^20^ consistent with the National TB Prevalence Survey in 2012.^25^

The gender imbalance observed in this study may be attributed to variances in health-seeking behavior, with females exhibiting a higher tendency to seek health services for TB symptoms.^26^ This inclination may be linked to the proactive health-seeking behavior of females, resulting in more cases being presented for screening through PCF. Men’s pursuit of their breadwinner roles and cost considerations strongly influence their willingness to seek care, and women’s willingness to seek care early is motivated by the need to preserve their caregiver roles and the protection of their children and families from illness. The gender ratio in this study and previous ones support the need for more ACF efforts for TB to help find cases among poor health-seeking males, especially in hard-to-reach communities. It further suggests a need for male gender-targeted intervention strategies to diagnose missing cases. This will help to break the chain of transmission, halt the disease, and avert catastrophic spending on health among the poor dwellers in those hard-to-reach areas.

Overall, about one-quarter of the TB cases were clinically diagnosed, and clinical diagnosis was highest among those aged 0–4 years and household contacts of Bac+ cases, respectively. This high proportion identified by clinical diagnosis may be due to the screeners’ high index of suspicion for TB and the employment of other supportive investigations like chest radiographs in these special groups. Bac+ TB cases are very infectious because droplet nuclei are generated when such persons cough, sneeze, shout, or sing.^27^ In contrast, the case definition of TB in children is difficult, and the diagnosis of childhood TB is a challenge, notably in those younger than 5 years.^28^ These young children most often cannot produce sputum for laboratory diagnosis, favoring more frequent clinical diagnosis with history, often supported with a chest radiograph.

As expected, the number needed to screen to diagnose 1 case of TB in our study was as low as 38 among household contacts of Bac+ TB cases and was as high as 441 overall and even higher (499) among those screened in the community. Also, household contacts of Bac+ TB cases yielded the highest prevalence of TB cases. The high prevalence of TB among household contacts could be influenced by factors such as close and prolonged exposure within households, shared living spaces, and common risk factors among individuals in the same household. Possible reasons for this observation could include increased transmission dynamics in confined living conditions and delayed diagnosis leading to prolonged exposure. Bearing the high infectivity of Bac+ TB cases in mind, screening among their contacts should be very meticulously carried out to detect all cases. However, caution must be exercised to reduce false positive results and prevent situations that might falsely appear as treatment failure; individuals who undergo empirical treatment for TB without laboratory evidence may experience lower survival rates compared to those with confirmed laboratory evidence.^29^ This necessitates that systematic screening for comorbidities, prompt diagnosis, and management of other infections be carried out in clinically diagnosed cases.^30^^,^^31^

Second to the highest proportion of TB cases among the screened populations is PLWH. It is established that PLWH are 20 times more likely than those without HIV to become sick with TB because HIV weakens the immune system, which makes it harder for the body to fight TB bacilli.^32^^,^^33^ HIV is a known driver of TB burden in Nigeria,^13^ and a higher prevalence of 22.6% of HIV-TB coinfection was reported in TB ACF among urban slums in southern Nigeria.^34^ All PLWH should be screened for TB, and those identified should be treated and given isoniazid preventive therapy for those eligible.

Commendably, 98.1% of the TB cases detected were started on treatment. Because Nigeria is one of the 10 high TB burden countries that had a worryingly low level of treatment coverage below 50% in 2021,^3^ there is a pressing need to improve on the situation if Nigeria and the world are to meet the goal of the End TB Strategy.^4^ Fortunately, community-based TB case-finding activities have continued in many of the communities involved in this project after the end of funding from TB REACH. The empowerment of the local stakeholders through this project has continued to drive those activities. Consequently, the Nigeria NTBLCP has adopted the measures used in this project and included them in the Global Fund-assisted programs aimed at expanding the strategies to scale up TB case detection in the country. In light of this, the NTBCPs in high TB burden countries can prioritize similar ACF strategies to upscale TB case detection, especially in hard-to-reach areas with high-risk populations where the routine health facility-based PCF approach is inadequate for meeting the goal of ending the TB epidemic.

The NTBCPs in high TB burden countries can prioritize similar ACF strategies to upscale TB case detection, especially in hard-to-reach areas with high-risk populations where routine health facility-based PCF is inadequate.

The execution of this project was fraught with several challenges. These included the high cost of water transport, poor access to diagnostic (X-ray and GeneXpert MTB/RIF) facilities in the affected areas, difficulties with diagnosis of childhood TB, and inadequate human resources for health in the hard-to-reach riverine communities.

It is important to note that these limitations may have influenced this program’s performance, making it necessary to acknowledge them when interpreting and applying the program’s outcomes. However, the difficulties posed by the high cost of water transport and the dearth of X-ray facilities were addressed through negotiations with the association of boat owners and proprietors of the X-ray facilities in the areas. By appealing to their sense of social responsibility to their communities and guaranteeing them an agreed number of trips per week, the project implementers achieved highly reduced transport fares for the project staff. Similarly, negotiations were held with the proprietors of the X-ray facilities in the LGAs. To address the human resources for health gap, the project identified and engaged medical doctors who resided in the areas, informed them of the project goal, and oriented them on the NTBLCP’s diagnostic algorithm for childhood TB. Review meetings with the medical doctors were held at appropriate intervals during the project. Difficult cases were shared on a designated WhatsApp platform where the NTBLCP Childhood TB focal persons, selected pediatricians, and the medical advisers of GLRA met to discuss the cases and advise on how to proceed.

Although this article emphasizes the successful outcomes of the intervention in terms of TB case notification, we acknowledge that the sustainability of ACF in the general context, and particularly in the ad hoc arrangements in this intervention, may not be guaranteed in the long term because of the costs associated with the project, especially in the African setting. We anticipate that gaining a comprehensive understanding of these implementation issues, including both the costs tied to the project and the ad hoc arrangements, as well as the opportunities they present, will significantly contribute to making well-informed decisions. In the meantime, diagnostic facilities need to be expanded to hard-to-reach areas with limited access to health facilities and PCF and the NTBLCP needs to build capacity to ensure the sustainability of the TB case-finding efforts.

Further, whereas this study provides valuable insights into TB control efforts in Nigeria from 2017 to 2020, researchers, policymakers, and practitioners should critically evaluate the relevance and applicability of our findings in the context of the evolving global health landscape and the specific circumstances prevailing after 2020. The COVID-19 pandemic may have influenced TB diagnosis, treatment, and reporting, potentially impacting this study’s findings.

### Limitations

We recognize that relying on signs and symptoms for TB screening and clinical diagnosis without laboratory confirmation introduces a risk of inaccuracies, given the potential overlap of these symptoms with those of other respiratory conditions. Nevertheless, measures were taken to address these concerns. Local clinicians and medical officers underwent comprehensive training and retraining in TB diagnosis, utilizing X-rays and clinical examinations to mitigate potential inaccuracies associated with clinical diagnosis.

## CONCLUSION

This ACF intervention in hard-to-reach riverine areas of Nigeria demonstrated a significant positive impact on TB case notifications, more than doubling the quarterly reporting rates. The study revealed a higher period prevalence than the national average, emphasizing the need for intensified ACF efforts in high-risk populations. The gender differences in TB cases detected, with a predominance of males, suggests a need for male gender-targeted interventions. Household contacts of Bac+ TB cases and PLWH are a key and vulnerable population for TB, and screening among them should be intensified and meticulously carried out to detect all cases. Challenges, including cost constraints and limited health care access, were addressed through innovative solutions, contributing to the success of the project.

Given these successful results, we recommend prioritization of resources to support the continuation of community-based TB case-finding and integration of successful ACF strategies into national programs, especially in hard-to-reach areas with high-risk populations, to address TB more comprehensively. We propose the following measures for policymakers: allocating resources for the continuation and expansion of community-based TB case-finding; integrating successful ACF strategies into national programs, with a specific focus on hard-to-reach areas; and enhancing diagnostic infrastructure in these areas to ensure widespread access to critical tools such as X-ray and GeneXpert MTB/RIF facilities. Emphasizing the urgent need for capacity-building initiatives will be pivotal for the sustainable success of TB control efforts in these challenging contexts.

## Supplementary Material

GHSP-D-23-00164-supplement.pdf

## Notes

**Peer Reviewed**

**First published online:** January 30, 2024.
