# Supplementary material for: Improving TB Case Detection Through Active Case-Finding: Results of Multiple Intervention Strategies in Hard-to-Reach Riverine Areas of Southern Nigeria
Source: Glob Health Sci Pract. 2024 Feb 28;12(1):e2300164. doi: 10.9745/GHSP-D-23-00164 (PMC10906553; doi:10.9745/GHSP-D-23-00164)
Supplement: GHSP-D-23-00164-supplement.pdf [file GHSP-D-23-00164-supplement.pdf]

Supplement to: Chukwu JN, Onah CK, Ossai EN, et al. Improving TB case detection through active case-finding: results of multiple intervention strategies in hard-to-reach riverine areas of southern Nigeria. *Glob Health Sci Pract*. 2024;12(1):e2300164. https://doi.org/10.9745/GHSP-D-23-00164

SUPPLEMENT TABLE. Result of Active Case Finding for TB Implemented in 15 Hard-to-Reach Riverine Local Government Areas in Southern Nigeria, Disaggregated by Intervention Strategy and Year of Report

Intervention Strategy	Performance Indicator	Output per Year				Total Output
		2017	2018	2019	2020	
Community outreach	No. of individuals screened for TB	12,676	92,527	584,518	200,754	890,475
	No. of presumptive TB identified	1,610	3,046	9,902	4,486	19,044
	No. of presumptive TB tested for TB	1,153	2,217	9,134	4,194	16,698
	No. of all forms of TB diagnosed	124	237	891	534	1,786
	No. of Bac+ DS-TB diagnosed	109	186	634	367	1,296
	No. of clinically diagnosed DS-TB	11	37	238	164	450
	No. of DR-TB diagnosed	4	14	19	3	40
	No. of Bac+ TB patients started on treatment	104	187	631	370	1,292
	No. of all forms of TB started on treatment	115	222	877	534	1,748
Health facility screening	No. of individuals screened for TB	3,484	12,477	97,494	56,547	170,002
	No. of presumptive TB identified	353	563	2,341	1,303	4,560
	No. of presumptive TB tested for TB	279	529	2,235	1,215	4,258
	No. of all forms of TB diagnosed	32	84	229	160	505
	No. of Bac+ DS-TB diagnosed	31	72	164	126	393
	No. of clinically diagnosed DS-TB	0	9	61	26	96
	No. of DR-TB diagnosed	1	3	4	8	16
	No. of Bac+ TB patients started on treatment	31	73	163	130	397
	No. of all forms of TB started on treatment	31	83	226	158	498
Screening among HIV-positive people	No. of individuals screened for TB	362	2,922	12,337	8,409	24,030
	No. of presumptive TB identified	14	70	220	92	396
	No. of presumptive TB tested for TB	10	53	216	90	369
	No. of all forms of TB diagnosed	3	11	33	10	57
	No. of Bac+ DS-TB diagnosed	2	6	27	7	42
	No. of clinically diagnosed DS-TB	1	5	6	3	15
	No. of DR-TB diagnosed	0	0	0	0	0
	No. of Bac+ TB patients started on treatment	1	6	26	7	40
	No. of all forms of TB started on treatment	2	11	33	10	56
Household contacts of Bac+ TB	No. of individuals screened for TB	54	176	2,816	1,576	4,622
	No. of presumptive TB identified	51	40	452	259	802
	No. of presumptive TB tested for TB	51	40	381	229	701
	No. of all forms of TB diagnosed	7	9	43	64	123
	No. of Bac+ DS-TB diagnosed	1	3	13	28	45
	No. of clinically diagnosed DS-TB	6	6	28	36	76
	No. of DR-TB diagnosed	0	0	2	0	2
	No. of Bac+ TB patients started on treatment	1	3	15	28	47
	No. of all forms of TB started on treatment	7	9	43	64	123
	No. of individuals screened for TB	54	176	2,816	1,576	4,622
	No. of presumptive TB identified	51	40	452	259	802

Abbreviations: Bac+, bacteriologically confirmed; DS-TB, drug-sensitive TB; DR-TB, drug-resistant TB.
